# Pharmacoeconomic Analysis of Medicines Used for Bronchial Asthma in Children in Kazakhstan

**DOI:** 10.34763/jmotherandchild.20252901.d-24-00046

**Published:** 2025-05-24

**Authors:** Elmira Serikbayeva, Nizom Suyunov, Baurzhan Makhatov, Ainash Atimtaikyzy, Aigul Ibragimova, Maksuda Abdullaeva

**Affiliations:** Department of Organization of Management and Economics of Pharmacy and Clinical Pharmacy, Asfendiyarov Kazakh National Medical University, Almaty 050000, Republic of Kazakhstan; Department of Organization of Pharmaceutical Business, Tashkent Pharmaceutical Institute, Tashkent 100015, Republic of Uzbekistan; Center for Continuous Professional Development, Shymkent 160011, Republic of Kazakhstan; Department of Pharmaceutical Disciplines, Astana Medical University, Astana 010000, Republic of Kazakhstan; Department of Pharmacology, Pharmacotherapy and Clinical Pharmacology, South Kazakhstan Medical Academy, Shymkent 160019, Republic of Kazakhstan; Department of Allergology, Republican Scientific Specialized Allergology Center, Tashkent 100109, Republic of Uzbekistan

**Keywords:** Exacerbation of asthma, economic assessment of asthma treatment, pharmacotherapy, drug safety, accessibility of therapy for children with asthma

## Abstract

**Background:**

This study aimed to calculate a pharmacoeconomic indicator, specifically the cost-effectiveness coefficient, for treating paediatric bronchial asthma with combined regimens of bronchodilators and inhaled corticosteroids.

**Material and methods:**

This study involves 54 children aged 6 to 12 years, who were divided into 6 groups depending on the age and severity of bronchial asthma. Treatment effectiveness is calculated by subtracting the percentage difference between exacerbation frequency and the number of patients. The calculation of pharmacoeconomic data was conducted using the cost-effectiveness ratio (CER).

**Results:**

For the treatment of mild bronchial asthma, the drug Berodual is used for inhalation through a nebuliser, moderate therapy is conducted using a combination of Flixotide and Salbutamol, and severe is stopped by a combination of Symbicort and Salbutamol. From the results obtained, notably, the CER for mild severity was 0.077 for children aged 6–8 years and 0.171 for the age group 9–12 years; for moderate severity, the CER values were 0.27 for the group 6–8 years and 0.35 for the category 9–12 years; severe asthma had the following indicators: 0.506 and 0.798 for groups aged 6–8 and 9–12, respectively.

**Conclusion:**

This study’s results indicate that the most cost-effective treatment regimen is in the age groups of 6–8 years. However, the calculation of drug dosages directly depends on the patient’s age and the severity of the disease. Further actions in scientific works should be directed to conducting empirical, statistical studies in the field of pharmacoeconomics of bronchial asthma among children from the standpoint of the state.

## Introduction

Bronchial asthma is a chronic lung disease characterised by an inflammatory process in the bronchial tree, a spasm of the smooth muscles of the respiratory tract, which leads to difficulty breathing. A person of any gender and age may suffer from bronchial asthma. Its symptoms include coughing, mainly at night, remotely audible wheezing on exhalation, and in more severe cases, inhalation, a feeling of pressure in the chest, which leads to difficulty breathing deeply and shortness of breath during exercise or at rest.

Untreated asthma can disrupt sleep and attention, lead to physical fatigue, and in the case of complications and deterioration, chronic respiratory failure or even death may develop. In addition, a patient with bronchial asthma has high financial costs for treatment since this disease is incurable and requires constant medication. In addition, more severe forms of bronchial asthma can lead to hospitalisation of a person and, in the most complicated cases, death. The prevalence of asthma in the world, according to the World Health Organisation [1], is about 339 million people and 455 thousand asthma-related deaths [2]. However, there are many treatment regimens for asthma, depending on the stage of the disease, the age of the patient, the course of the disease, and other factors. In this regard, researchers are actively investigating the issue of pharmacoeconomics of drugs to identify the most profitable and effective treatment regimen for bronchial asthma, as well as other problems in this area.

According to statistics, in Kazakhstan, the prevalence of clinical bronchial asthma registered by a doctor is 2.5%, asthma that has been treated is 2.57%, and remote wheezing was observed in 4.98% of patients, however, there is very little statistical data [3]. It is known from world statistics that, on average, the prevalence of bronchial asthma in children aged 6–7 years is about 16%, and in the age group of 13–14 years about 10% [4]. In addition, it is noted that in 50% of cases, bronchial asthma remains without adequate treatment and control due to a large number of factors and causes, despite the fact that advances in pharmacotherapy and the dissemination of guidelines on this subject are at a high level.

The manifestation of asthma may be different for each person since not all patients have the same picture of inflammation, which can lead to different clinical symptoms and, as a result, different therapeutic regimens. Patients often suffer from seizures, which can occur from several cases per day to several cases per year. Deterioration of the condition usually occurs at night or during physical activity. An asthma attack can provoke acute respiratory illness, seasonal weather changes, the influence of dust and allergens, tobacco smoke, the flowering of herbs and trees, animal hair or bird feathers, flavoured cosmetics, and household chemicals [5].

The causes of bronchial asthma include a large number of factors. These include:

- an increase in the likelihood of the disease in the case of bronchial asthma in the closest relative in the family [6];
- the presence of another allergic disease in the patient—atopic dermatitis, allergic rhinitis;
- the incidence of asthma increases in people living in more developed regions; low birth weight, prematurity, exposure to polluted air including tobacco, and frequent respiratory diseases;
- exposure to the most common allergens, the presence of dust mites and mould, air pollution in residential premises, contact with chemicals also increases the risk of asthma [7];
- and high chances of developing bronchial asthma exist in overweight or obese people and children.

In addition, the increase in the incidence and prevalence of bronchial asthma in children is substantially increasing in areas with low income and increased urbanisation [8,9]. In paediatric practice, the diagnosis of bronchial asthma is established mainly “clinically”, that is, based on the manifestation of respiratory symptoms, clinical history, frequency of exacerbations, and signs of variable reversible restriction of airflow, which can be determined using a functional lung test with an assessment of bronchiolitic response.

One of the main principles of the state policy in the field of circulation of medicines aimed at protecting the health of citizens is the formation of a state policy in the field of circulation of medicines that meets the needs of the healthcare system [10,11], the development and support of national production of medicines [12,13], and ensuring transparency and integrity of public administration and business in the field of circulation of medicines [14,15].

Given the limited resources of the healthcare system, it is important to manage them correctly and allocate funds effectively. With the help of pharmacoeconomics analysis, it is possible to determine the most cost-effective treatment methods that will help use the budget with maximum efficiency and minimum expenditure of funds, which will allow more patients to receive high-quality medical care. The determination of pharmacoeconomic indicators can help the doctor and the patient make the right decision in choosing a treatment that allows them to achieve the best results at minimal cost. This allows for improvement to the patient’s quality of life and financial situation.

Pharmacoeconomic analysis is divided into several categories [16]:

1. Direct medical costs, which mainly include financing from the budget to ensure diagnosis and treatment in public institutions and the cost of treating the patient themselves.
2. Direct non-medical costs, which cover the costs of transporting the patient to emergency care facilities and spending on a specific diet, if required.
3. Indirect costs for society and the patient, which include the loss of time at work, financial losses of an employee due to disability, and payment of time spent on sick leave, including caring for a sick child, skipping school days.
4. Intangible expenses that affect the quality of life of the patient.

Five economic methods of analysing drug therapy are used to calculate the pharmacoeconomic indicator: “cost-minimisation analysis” (CMA), “cost-effectiveness ratio” (CER), “cost-benefit analysis” (CBA), “cost-utility analysis” (CUA), and “willingness to pay”. The calculation of the CMA allows for comparing the costs of alternative treatments for drug therapy, provided that these methods are effective in accordance with standard treatment regimens. With this method, the least expensive treatment method, comparing new drugs with generics, can be chosen. If, when using this type of pharmacoeconomic analysis, equivalent clinical results have not been proven, then other methods are resorted to. The CER analysis compares the financial costs and the results of alternative treatments, which are calculated in “natural” terms. “Natural” indicators are the frequency of exacerbations over a certain period of time, the number of days without symptoms of the disease, and an increase in life expectancy. This method allows for the determination of which treatment regimen leads to a good effect for the least expenditure of finances. A negative or lower value indicates that the treatment method has an increase in efficiency and a decrease in costs [16].

The calculation of the CBA indicator evaluates the costs and results of treatment by determining the greatest benefit or benefit to the cost of alternative treatment options. The CBA differs from the CER in that the results of this analysis are produced in monetary terms, that is, it is possible to compare different health programmes in monetary units. The calculation of CUA is similar to CER since it allows for the determination of which alternative treatment method leads to the result that is most suitable for the patient in terms of unit cost. This analysis allows for comparing the financial cost with the results of other treatment methods in utilitarian terms and considering the effectiveness of medical treatments not by one but by several factors. “Willingness to pay” is a way to consider quantifying the benefits of health in monetary terms. This indicator is estimated based on data from surveys and questionnaires of patients who are willing to pay for certain achievements in improving their health. According to world statistics, the most commonly used indicators are CER – 50%, CMA – 30%, CBA – 15%, and CUA – 5% [16].

Studies on direct medical expenses per patient per year were conducted by Wu et al. [17], who established that in children aged 0 to 14 years, the cost of treating bronchial asthma is USD 75, however, about 60% of medical expenses were covered by insurance. The highest rates were the use of antibiotics in large cities in almost 100% of patients, in children aged 3 years and younger – 63.6%, and in cities of the fourth and fifth levels – 77.1%. Outpatient clinics were visited in 98.58% of cases, level 3 hospitals were 62.08% and general hospitals were visited most often, at 72.72%. This indicates that the cost of medical care for bronchial asthma is calculated not only according to the costs of the patients themselves but also according to the use of other drugs, the age of the patient, the severity of the case, and the visit to a medical institution.

Finkelstein et al. [18] examined the economic costs of treating bronchial asthma in adults and children. Using an online cross-poll, researchers examined patients’ requests for medical help, the number of days of treatment, absences at work, decreased performance and productivity due to symptoms of bronchial asthma. Further, these values were calculated and monetised to analyse the total cost of treatment. Thus, for the annual treatment of bronchial asthma in children, USD 0.25 billion is needed, while 64% of the cost is spent on an uncontrolled group, 26% on a partially controlled group, and 10% on a well-controlled group. The total cost of asthma treatment in children and adults amounted to USD 1.5 billion, with almost 80% attributed to disability and productivity.

Nurmagambetov et al. [19] conducted a study from a national standpoint to assess the complex costs of treating bronchial asthma, considering medical expenses, the cost of missed work and school days, mortality, in addition, race/ethnic origin, financial situation, insurance status, and education were considered. In the course of the study, it was determined that the annual expenditure on the treatment of bronchial asthma per person is USD 3,266, of which USD 1,830 was spent on medicines, USD 640 – on visits to outpatient centres, USD 530 – on hospital treatment, USD 176 – on outpatient visits to the hospital, and USD 105 – on treatment in the department emergency care. In addition, it was noted that skipping work and school cost USD 3 billion. Perry et al. [20] examined the direct costs of treating bronchial asthma in children. The average annual expenditure per child ranged from USD 3,080 to USD 13,620, for drug costs between from USD 1,030 to USD 2,120, for the cost of hospital treatment between USD 340-2020, and for outpatient treatment between USD 1,050 to USD 8,040.

Chen et al. [21] examined the financial costs of a combination of bronchial asthma and other concomitant diseases, where it was established that half of the cost of asthma treatment accounted for concomitant diseases. Rank et al. [22] considered the indicator of appropriate use of medicines for the treatment of asthma and determined that the average for children who suffered from a stable form of asthma was 0.35 instead of the target value of 0.7. These results suggest that the proper use of medicines for the treatment of bronchial asthma will help improve the prognosis and course of the disease, reduce variability, and enhance the importance of healthcare. In addition, in a study by Chastek et al. [23], according to the examination of waste for the treatment of persistent and severe asthma, it was established that patients with persistent bronchial asthma who had two or more episodes of exacerbation were two times more likely to use drugs to stop seizures, compared with patients with severe asthma. In addition, spending on the treatment and monitoring of bronchial asthma was also three times higher in patients with persistent form compared to those with severe bronchial asthma.

Tabyshova et al. [24] examined the prevalence and economic aspects of respiratory diseases in Central Asia. However, no data on the cost of treatment in Kazakhstan were found, and estimated information was described on direct costs for primary medical care for bronchial asthma, which amounted to USD 288.9, as well as for secondary medical care—USD 914.3 in Russia. In addition, the level of knowledge, skills and commitment to treatment in patients with bronchial asthma in Kazakhstan remains extremely low, and treatment with medicines is far from optimal [25].

The purpose of this study was to calculate the pharmacoeconomic value of some drugs using the CER.

## Materials and methods

This paper was based on the pharmacoeconomic calculation of medicines for the treatment of bronchial asthma. Before starting the study, the parents of children who were observed by a paediatrician and a pulmonologist with bronchial asthma gave written consent to participate in the study, in accordance with the standards of the Helsinki Declaration [26]. After that, the data of outpatient records of patients with confirmed bronchial asthma of mild, moderate, and severe severity in the age group from 6 to 12 years were analysed. 54 children were divided into 2 groups by age: from 6 to 8 years (32 children) and from 9 to 12 years (22 children). Each group was distributed considering the severity of asthma (Table 1).

**Table 1.** Distribution of patients according to severity, age.

**Degree of severity**	**Age**	**Number of people**
Mild	From 6 to 8 years old	10
	From 9 to 12 years old	8
Medium	From 6 to 8 years old	12
	From 9 to 12 years old	9
Severe	From 6 to 8 years old	10
	From 9 to 12 years old	5

*Source:* compiled by the authors.

After a thorough investigation of the patient data and a number of consultations, all participants received basic treatment regimens for bronchial asthma, in which the following drugs were used: salbutamol inhalers to stop asthma attacks, Berodual nebuliser inhalation solution (ipratropium bromide + phenoterol), Flixotide inhalers (fluticasone propionate), and Symbicort (budesonide + formoterol) to etiological anti-inflammatory effect. There were six treatment regimens used that corresponded to the stages of bronchial asthma and the age group. The study did not consider gender, allergic history, family composition, housing conditions, and environmental conditions.

Treatment in children was conducted based on the severity of bronchial asthma, according to the dosage of the drug for each age group (Table 2). For the therapy of a mild form, a solution of Berodual was used for inhalation through a nebuliser. Two treatment regimens were used:

- Berodual of 1 ml + 4 ml of saline solution, 20 days, the cost of this scheme was USD 5.4 (2380 tenge);
- and Berodual of 2 ml + 4 ml of saline solution, 1 time per day, 20 days, the cost of this scheme was USD 10.8 (4760 tenge).

**Table 2.** Treatment regimens depending on the stage, age.

**The stage of bronchial asthma**	**Age**	**Medication**	**Cost USD (tenge)**
The mild stage	6–8 years	Berodual inhalation solution 20 ml 1 fl	USD 5.4 (2380 tenge)
	9–12 years	Berodual inhalation solution 20 ml 2 fl	USD 10.8 (4760 tenge)
The medium stage	6–8 years	Flixotide 50 mcg 120 doses 1 fl + Salbutamol 100 mcg 200 doses 1 fl	USD 11.17 (4920 tenge)
	9–12 years	Flixotide 50 mcg 120 doses 2 fl + Salbutamol 100 mcg 200 doses 1 fl	USD 19.61 (8640 tenge)
The severe stage	6–8 years	Symbicort 80/4.5 mcg 120 doses of 1 fl. + Salbutamol 100 mcg 200 doses of 1 fl.	USD 25.3 (11150 tenge)
	9–12 years	Symbicort 80/4.5 mcg 120 doses 2 fl. + Salbutamol 100 mcg 200 doses of 1 fl.	USD 47.88 (21100 tenge)

*Note*: The current exchange rate is USD/KZT – 1/528.02.

*Source*: compiled by the authors.

The dosage was calculated according to the age of the child.

The drug control for moderate severity of bronchial asthma consisted of a combination of Salbutamol and Flixotide. Two treatment regimens were applied:

- Salbutamol 100 mcg by 1–2 inhalations during seizures, that is, on demand + Flixotide 50 mcg 1 dose 2 times a day, 1 month, then Flixotide 50 mcg 1 dose 1 time a day, 2 months. The cost of treatment in this case was USD 11.17 (4920 tenge);
- and Salbutamol 100 mcg 1–2 inhalations on demand + Flixotide 50 mcg 2 inhalations 2 times a day, 1 month, then Flixotide 50 mcg 1 inhalation 2 times a day, 2 months, which cost USD 19.61 (8640 tenge).

The treatment of severe asthma in children was performed with a combination of drugs Symbicort + Salbutamol. The use of Symbicort 80/4.5 mcg for 1 inhalation 2 times a day, 1 month, then 1 inhalation 1 time a day, 2 months + Salbutamol 100 μg on demand, which amounted to USD 25.3 (11,150 tenge). In turn, Symbicort 80/4.5 mcg for 2 inhalations 2 times a day, 1 month, then 1 inhalation 2 times a day, 2 months + Salbutamol 100 μg on demand, cost USD 47.88 (21,100 tenge). The cost of one bottle was: Berodual 20 ml inhalation solution – USD 5.4 (2,380 tenge), Salbutamol 100mcg 200 doses – USD 2.27 (1,200 tenge), Flixotide 50 mcg 120 doses – USD 8.44 (3,720 tenge), and Symbicort 80/4.5 mcg – USD 22.58 (9,950 tenge).

During the course of treatment and after its completion during the year, patients and their parents filled out a questionnaire where they noted the number of exacerbations of bronchial asthma per year. According to these data, the frequency of exacerbations was calculated as a percentage in each group. Following this, the effectiveness was calculated by subtracting the difference in the frequency of exacerbations from the number of patients as a percentage [16]. The CER (1) was used to select the most effective treatment regimen from the standpoint of pharmacoeconomics:

(1) CER=DC/EF,

where CER indicates the costs per unit of effectiveness; DC is the direct cost (cost of treatment); and Ef is the effectiveness of treatment (percentage of cured patients).

## Results

Bronchial asthma in children is a serious problem for children and their parents, which affects not only the financial side of each family but also the loss of working capacity, leading to a less active lifestyle and constant struggle with frequent exacerbations. Based on this, researchers have conducted a large number of studies on the subject of the treatment of bronchial asthma. However, due to the existence of a large number of health systems and different treatment regimens, there is a need to adapt treatment approaches to local conditions in each country. In addition, data on the cost of treatment for bronchial asthma with the most effective treatment regimens and minimal financial expenses that correspond to a specific country, region, or city are needed for each health care system [27].

In the field of public health, a patient suffering from bronchial asthma who has been left without proper monitoring and treatment can cause a large number of unplanned outpatient visits to a medical institution, additional consultations with narrow specialists, seeking medical help in emergency departments and treatment in an inpatient department. This situation leads to high financial costs for the healthcare system, society, and the patients themselves, which can be avoided by prescribing adequate therapy with the most cost-effective treatment regimen. The lack of control also leads to insufficient diagnosis, irregular follow-up, violating the standard treatment regimen and abuse of drugs for the relief of urgent conditions, such as salbutamol. In addition, the number of medicines is growing every year, and the cost of drugs remains high [16]. Limited funding from the state and large expenditures of money on the part of a patient suffering from bronchial asthma require optimisation and improvement of the quality of treatment, identification of new effective technologies and schemes in evidence-based medicine that will have the lowest costs for the treatment, prevention, and recovery of patients with asthma.

Among the main available medicines for the treatment of bronchial asthma in children in Kazakhstan, it is possible to note the combined drugs Berodual, which includes ipratropium bromide [28] and fenoterol [29]; salbutamol [4], flixotide [30], symbicort, which includes budesonide and formoterol [31], which were prescribed to patients in the present study.

The number of exacerbations that patients noted during the year from the start of treatment, according to the compiled schemes in each group, was expressed in the following data (Table 3): In the mild stage of bronchial asthma in children aged 6–8 years, up to 3 exacerbations per year were noted, the age group of 9–12 years had the same data – up to 3 exacerbations in the treatment of moderate severity, children aged 6–8 years had up to 6 exacerbations, and children aged 9–12 years – had up to 4 exacerbations; in the treatment of severe severity, up to 5 exacerbations were noted in the age group of 6–8 years, and in the group of 9–12 years – up to 2 exacerbations per year.

**Table 3.** The number of exacerbations, according to the treatment regimens in each group.

**The stage of bronchial asthma**	**Age group**	**Number of patients**	**Treatment regimen**	**The frequency of exacerbations per year**	**Percentage ratio**
The mild stage	6–8 years old	10	Scheme 1	3	30%
	9–12 years old	8	Scheme 2	3	37%
The medium stage	6–8 years old	12	Scheme 1	6	60%
	9–12 years old	9	Scheme 2	4	44%
The severe stage	6–8 years old	10	Scheme 1	5	50%
	9–12 years old	5	Scheme 2	2	40%

The effectiveness for each group was determined by calculating the percentage difference between the number of treated children with bronchial asthma and the number of reported cases of exacerbation per year in these patients. Thus, for the group with mild severity, the effectiveness of treatment according to scheme 1 was 70%, and according to scheme 2 – 63%; for moderate severity, scheme 1 – 40%, scheme 2 – 56%; for severe severity, scheme 1 – 50%, scheme 2 – 60%.

As a result of calculations of the “cost/effectiveness” coefficient in pharmacoeconomic aspects in the treatment of bronchial asthma, it was determined that the cost of basic treatment regimens, considering specific age groups and with various degrees of severity of bronchial asthma, ranges from USD 5.4 (2380 tenge) to USD 47.88 (21100 tenge), and the effectiveness of the treatment regimens used varies from 40% up to 70%. Based on this, the CER coefficient was calculated for each severity of bronchial asthma and age group (Table 4).

**Table 4.** Calculation of CER coefficients for different severity levels of bronchial asthma.

**Severity**	**Treatment tactics**	**The cost of the course, USD (tenge)**	**Effectiveness, %**	**CER**
Mild	Scheme 1	USD 5.4 (2380 tenge)	70	0.077 (34)
	Scheme 2	USD 10.8 (4760 tenge)	63	0.171 (75.5)
Moderate	Scheme 1	USD 11.17 (4920 tenge)	40	0.27 (123)
	Scheme 2	USD 19.61 (8640 tenge)	56	0.35 (154.2)
Severe	Scheme 1	USD 25.3 (11150 tenge)	50	0.506 (223)
	Scheme 2	USD 47.88 (21100 tenge)	60	0.798 (351.6)

*Source:* compiled by the authors.

The calculation of the pharmacoeconomic indicator using the cost-effectiveness coefficient is conducted to determine the lowest value of this indicator, however, notably, the calculation of dosages depends on many factors, one of which is age. Thus, the older the child is, the higher the dosages of prescribed medicines will be, which will lead to an increase in the cost of treatment and, as a result, an increase in the cost/effectiveness ratio. From the data obtained, the lowest CER level was determined in all age groups of 6–8 years for each severity, which was 0.077 (34) for mild, 0.27 (123) for moderate, and 0.506 (223) for severe. In addition, there was a correlation between the severity of bronchial asthma and the cost of treatment, that is, the more severe the asthma, the higher the CER index.

## Discussion

This study was conducted to determine the most effective and least costly treatment regimen among children with bronchial asthma using the CER coefficient. However, the results obtained had some disadvantages in the form of a small sample and the use of only three treatment regimens in two age groups with varying degrees of severity. The results show that the most effective and beneficial treatment was performed in children with mild severity in the age group of 6–8 years. Therewith, the calculation of dosages was conducted according to age, therefore, the cost of a course of treatment with any degree of severity in the age groups of 6–8 years was lower compared with the age groups of 9–12 years. In addition, the coefficient was calculated considering the frequency of exacerbations per year, and its values did not differ much but fluctuated between 40–70%, which also affected the result.

In the study by Rege et al. [32] on the control of bronchial asthma at the outpatient admission level, the data from the examination of children from 6 to 17 years old were considered. From the results, it is worth highlighting that, on average, almost 2.5 million visits with primary bronchial were registered per year. Data on the control and severity of this disease were noted in the documentation only in 36% and almost 34% of cases, respectively. Patients with certain ethnicities living in the regions under study, suffering from chronic sinusitis, who visited a doctor in the fall had a high chance of registering the disease with subsequent control. On the other hand, during visits in the spring, patients with unregistered asthma severity had a lower chance of asthma control. Moderate and severe asthma had a greater chance of developing into uncontrolled bronchial asthma compared to the persistent form, and visits in the summer had a lower chance of further control and effective treatment. This study did not consider regional indicators and nationality since it was conducted within the same region, and a small sample of patients was ineffective in analysing treatment results in relation to seasonal visits. Based on this study, it can be concluded that the problem of the pharmacoeconomics of bronchial asthma in children is much deeper since registration and control of the management of this disease in many countries, such as Kazakhstan, suffers greatly.

Pugliese et al. [33] calculated the cost of treatment of bronchial asthma with moderate and severe severity in emergency departments with acute conditions in the form of exacerbations and controlled types of asthma. The study selected 800 cases of bronchial asthma in children and adults and determined that adult patients had treatment costs from USD 350 to USD 860, and expenses for children ranged from USD 380 to USD 1,150, depending on the severity of the disease. These data confirm that the medium and severe stages of bronchial asthma play an important role in the life of the patient and society in general due to the chronic course, frequent exacerbations, disability, and a large number of requests for medical help. This indicates the great importance of conducting such studies in Kazakhstan, where the cost of treatment of bronchial asthma has not been examined.

In the theoretical study of Mensella et al. [34], all types of pharmacoeconomic aspects of the treatment of bronchial asthma were considered, where the costs of therapy and its consequences were compared, and the results of direct drug costs and seeking medical help were evaluated. From the results obtained by Mensella et al. [34], it should be emphasised that the CER of bronchial asthma are aimed at analysing the financial component of the treatment of this disease. However, the influences of therapy regimens, treatment time, and a high-quality cost database are important, which were ignored in many studies. Since bronchial asthma is a chronic disease that requires constant long-term treatment, and studies usually have a short time period, the CER data may not have completely reliable results. Therefore, due to various ineffective schemes, high variability of treatment methods, different pricing of medicines, and inadequate costs for bronchial asthma therapy, assessment of pharmacoeconomic effectiveness is quite a difficult task. In this study, the treatment time was three months, and the observation and registration of exacerbations were conducted during the year. Thus, for the CER indicator to be as reliable as possible, it is necessary to pay attention to the accuracy of phenotyping, determining the response to treatment, especially in patients who have frequent exacerbations, and use a longer follow-up period. Compared with this study, the paper of Mensella et al. [34] proves the importance of each factor influencing the CER indicator.

In addition, Zyryanov et al. [35] conducted a study on the financial costs of treating bronchial asthma using the combination drug budesonide/formoterol (*Symbicort Turbuhaler*) as a maintenance therapy compared with standard treatment regimens for mild, moderate, and severe severity using the combined drug salmeterol/fluticasone and salbutamol on demand. The study was conducted from the standpoint of the financial costs of the public health system. The results showed an increase in the number of patients receiving *Symbicort Turbuhaler* for many years, up to 5.5% with the mild stage, 7.7% with the medium stage, and 9.7% with the severe stage, hence an increase in the pharmacoeconomic index to USD 1300. The use of *Symbicort Turbuhaler* for any degree of bronchial asthma reduces the frequency of exacerbations and reduces the financial burden on the country’s budget and the healthcare system. The similarity of this study with the present one lies in the use of the combined drug *Symbicort Turbuhaler* (budesonide/formoterol), which has a positive effect on efficiency and financial costs.

An international study by Lane et al. [36] was based on the examination of the treatment of bronchial asthma at the level of primary and secondary medical care. An analysis of all asthma exacerbations showed that the country has a substantial impact on the cost of treating exacerbations. On average, the cost of secondary care therapy (USD 1,430) exceeded primary care (USD 480). The value of these indicators depended on the age and type of care. As the patient’s condition deteriorated, funding for secondary medical care grew rapidly, but the cost of primary medical care remained stable. The results of this study also show a correlation between the severity and the growth of the cost index due to frequent exacerbations. In the study by Lane et al. [36], it was shown that the costs of treating and monitoring bronchial asthma remain quite high, but treatment regimens have great potential to reduce the frequency of exacerbations and severity of the condition and increase the quality of life of the patient, which still leads to economic benefits. This study has similar results depending on the cost of treatment of bronchial asthma and the patient’s age. In addition, in the study by Halmai et al. [37], it was established that the quality of many pharmacoeconomic examinations had various variations and was somewhat uncertain due to the use of different methods and the relevance of some data since there is a lack of empirical pharmacoeconomic studies in the treatment of bronchial asthma in children. Therefore, researchers recommend using new technologies that will be maximally adapted to local health systems to maximise cost savings in the treatment of bronchial asthma in children.

## Conclusions

In this study, it was established that bronchial asthma is a chronic, incurable disease characterised by an inflammatory process in the bronchi, spasms of the smooth muscles of the pulmonary system, which leads to difficulty breathing, cough with distant wheezing mainly at night, a feeling of pressure in the chest. The prevalence of asthma among children worldwide ranges from 10% to 16%, and the registration of bronchial asthma in Kazakhstan varies from 2.57% to 4.98%. In this regard, the control of this disease worsens, which leads to an increase in cases of exacerbation and increases the costs of the patient, society, and state for treatment and prevention.

The calculation of the pharmacoeconomic indicator for three treatment regimens for bronchial asthma in children of two age categories with varying degrees of severity showed that the CER coefficient for mild, moderate, and severe severity with the most economic benefit was in the age group of 6–8 years and was 0.077, 0.27, and 0.506, respectively. However, the calculation of the dosage of drugs for the treatment of bronchial asthma was conducted considering the age of patients—therefore, the older the age, the higher the dosage of the drug, the cost of the course of treatment, and the higher the pharmacoeconomic indicator. In addition, there was a correlation between the severity of asthma and the cost of treatment, that is, the more severe the patient’s condition, the higher the CER coefficient.

The limitations of this study were a small sample of clinical cases and the appointment of only three treatment regimens for the age groups of 6–8 years and 9–12 years for mild, moderate, and severe severity levels. In addition, the lack of statistical data and empirical studies on the prevalence of bronchial asthma and pharmacoeconomics among children and adults in Kazakhstan complicates the development of this subject. In further research, it is necessary to direct efforts to examine the incidence of bronchial asthma in children and adults, determine the economic costs of outpatient and inpatient treatment, relief of acute conditions, financing associated with disability and skipping school days, and the determination of the most effective and cost-effective treatment regimens.

## Abbreviations

CER: Cost-Effectiveness Ratio
DC: Direct Cost
Ef: Effectiveness of Treatment
CMA: Cost-Minimisation Analysis
CBA: Cost-Benefit Analysis
CUA: Cost-Utility Analysis

## Key points

- Bronchial asthma is a chronic disease that causes breathing difficulties, with global prevalence in children at 10–16%.
- Asthma rates in Kazakhstan range from 2.57% to 4.98%, leading to poor control and higher treatment costs.
- Cost-effective asthma treatments were identified for children aged 6–8 years, with severity correlating to higher costs.
- Limited data on asthma in Kazakhstan calls for broader research on prevalence and treatment economics.
- Public health should focus on improving asthma management and closing data gaps to reduce costs and improve care.
